# Tryptophan metabolism is a physiological integrator regulating circadian rhythms

**DOI:** 10.1016/j.molmet.2022.101556

**Published:** 2022-07-29

**Authors:** Paul Petrus, Marlene Cervantes, Muntaha Samad, Tomoki Sato, Alina Chao, Shogo Sato, Kevin B. Koronowski, Grace Park, Yasmine Alam, Niklas Mejhert, Marcus M. Seldin, José Manuel Monroy Kuhn, Kenneth A. Dyar, Dominik Lutter, Pierre Baldi, Peter Kaiser, Cholsoon Jang, Paolo Sassone-Corsi

**Affiliations:** 1 Center for Epigenetics and Metabolism, U1233 INSERM, Department of Biological Chemistry, University of California, Irvine, Irvine, CA 92697, USA; 2 Institute for Genomics and Bioinformatics, Department of Computer Science, University of California Irvine (UCI), Irvine, CA, USA; 3 Department of Biological Chemistry, Chao Family Comprehensive Cancer Center, University of California Irvine, Irvine, CA 92697, USA; 4 Department of Medicine (H7), Karolinska Institutet, C2-94, Karolinska University Hospital, 141 86 Stockholm, Sweden; 5 Center for Epigenetics and Metabolism, Department of Biological Chemistry, University of California, Irvine, CA, USA; 6 Computational Discovery Research, Institute for Diabetes and Obesity (IDO), Helmholtz Diabetes Center (HDC), Helmholtz Zentrum Munich – German Research Center for Environmental Health, 85764 Neuherberg, Germany; 7 German Center for Diabetes Research (DZD), 85764 Neuherberg, Germany; 8 Metabolic Physiology, Institute for Diabetes and Cancer (IDC), Helmholtz Diabetes Center, Helmholtz Zentrum Munich – German Research Center for Environmental Health, 85764 Neuherberg, Germany; 9 Department of Biological Chemistry, School of Medicine, University of California Irvine, Irvine, CA, USA

**Keywords:** Circadian, Metabolism, Tryptophan, Systems biology

## Abstract

**Objective:**

The circadian clock aligns physiology with the 24-hour rotation of Earth. Light and food are the main environmental cues (zeitgebers) regulating circadian rhythms in mammals. Yet, little is known about the interaction between specific dietary components and light in coordinating circadian homeostasis. Herein, we focused on the role of essential amino acids.

**Methods:**

Mice were fed diets depleted of specific essential amino acids and their behavioral rhythms were monitored and tryptophan was selected for downstream analyses. The role of tryptophan metabolism in modulating circadian homeostasis was studied using isotope tracing as well as transcriptomic- and metabolomic- analyses.

**Results:**

Dietary tryptophan depletion alters behavioral rhythms in mice. Furthermore, tryptophan metabolism was shown to be regulated in a time- and light- dependent manner. A multi-omics approach and combinatory diet/light interventions demonstrated that tryptophan metabolism modulates temporal regulation of metabolism and transcription programs by buffering photic cues. Specifically, tryptophan metabolites regulate central circadian functions of the suprachiasmatic nucleus and the core clock machinery in the liver.

**Conclusions:**

Tryptophan metabolism is a modulator of circadian homeostasis by integrating environmental cues. Our findings propose tryptophan metabolism as a potential point for pharmacologic intervention to modulate phenotypes associated with disrupted circadian rhythms.

## Highlights

• Dietary tryptophan and threonine controls circadian behavioral rhythms.
• Tryptophan is metabolized in a time- and light- dependent manner.
• Light cycles and tryptophan intake coordinate temporal regulation of metabolism and transcription.
• Tryptophan metabolism regulate expression of the core clock machinery.

## Graphical abstract

**Figure:**
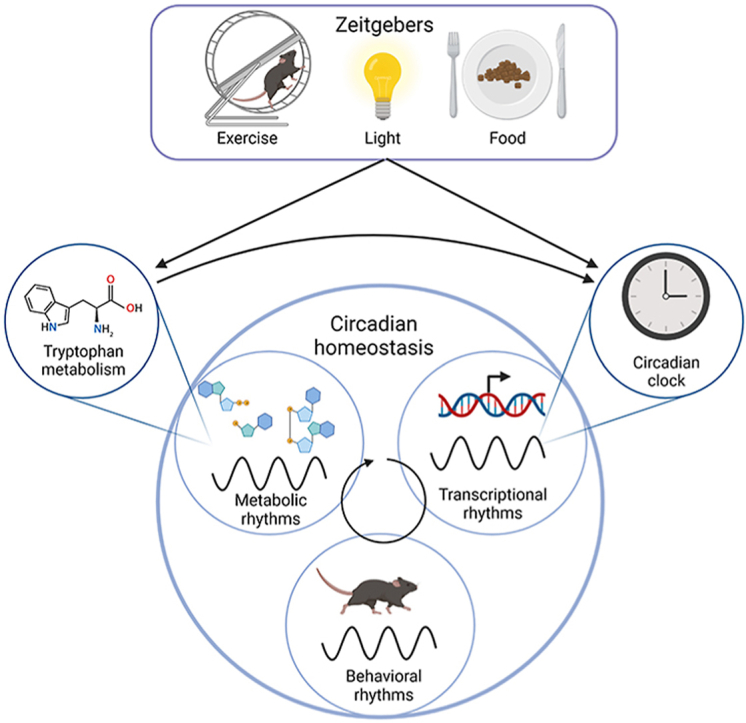

## 1 Introduction

Throughout the evolution of life on Earth, the planet has revolved around the sun while rotating around its own axis once every ∼24-h giving rise to day and night cycles. Thus, biological systems have adapted to predict these geophysical phenomena [1]. Modern societies, with 24-h access to artificial light and food, disrupt the synchrony between circadian homeostasis and day/night-cycles, contributing to several diseases including obesity, neurodegenerative disorders, and cancer [[2], [3], [4], [5], [6], [7]]. The circadian clock is an evolutionary conserved molecular circuit that allows living organisms to anticipate and adjust to daily environmental fluctuations [8]. At its core, the circadian clock is constituted of a transcriptional-translational feedback loop where the transcription factors BMAL1 and CLOCK heterodimerize and bind to E-box elements at the promotors of hundreds of genes including their own repressors *Per1-3* and *Cry1-2* [9]. The repressors accumulate in the cytosol and translocate to the nucleus to inhibit the transcriptional complex, a cycle that takes about 24 h. The core clock machinery is tightly coupled with metabolism that communicates environmental cues to regulate circadian transcription [10,11].

The clock is synchronized by environmental pacemaker cues termed *zeitgebers* with light being the most dominant signal [12]. Photic signals reset the master clock in the suprachiasmatic nucleus (SCN) located within the hypothalamus [13,14]. The SCN communicates photic cues to other clocks within the brain as well as to peripheral organs [[15], [16], [17]]. Feed-fasting cycles are also potent *zeitgebers* [2,[18], [19], [20]] that mainly regulate rhythms in peripheral tissues [[19], [20], [21], [22], [23], [24]]. However, the central clock is also influenced by nutrition [16,17,[25], [26], [27]]. In fact, nutritional challenges such as high fat diet reprogram behavioral, central-metabolic/transcriptional rhythms [28,29] as well as altering the SCN response to photic signals [30,31]. However, little is known about the *zeitgeber* properties of specific dietary nutrients such as amino acids that serve as signaling molecules in inter-organ communication [32].

Herein, we tested the necessity of each essential amino acid (EAA) for behavioral circadian rhythms and identified tryptophan as a central modulator of circadian homeostasis.

## 2 Material and methods

### 2.1 Mice

Male C57BL/6 mice (the Jackson Laboratory), aged 8 weeks, were acclimated to 12hr light/dark cycles or constant darkness prior to the intervention. Mice were group-housed during the acclimation period and were individually housed during the intervention period. All procedures were performed in accordance with guidelines of the Institutional Animal Care and Use Committee at the University of California, Irvine.

### 2.2 Intervention procedures

After the acclimation period mice were single housed for one week under 12-h light/dark cycles (LD) or in constant darkness (DD) followed by the introduction of the tryptophan depleted (-Trp, TD.130674), threonine depleted (-Thr, TD.190881), phenylalanine depleted (-Phe, TD.210439), methionine depleted (-Met, TD.140119), lysine depleted (-Lys, TD.210440), histidine depleted (-His, TD.190882) and branched-chain amino acid depleted (-BCAA, TD.210441) -diet and nitrogen-matched control diet (Ctrl, TD.01084) (Tekla diets). The dietary intervention was given *ad libitum* and continued for two weeks under the same lighting conditions, either 12-h light/dark cycles or in constant darkness. The jet-lag experiment was performed while mice were on the diet intervention and in a 12-h light/dark cycle. The light schedule was inverted by extending the dark period by 12-h on the 7th day. Supplementation of the tryptophan metabolic intermediates, kynurenine (K8625, Sigma) and 5-hydroxy-L-tryptophan (H9772, Sigma) was performed throughout the second week of the dietary intervention under constant darkness. Mice were injected intraperitoneally with saline or 20 mg/kg body weight of each compound which is within or below the amounts used in previous studies [[33], [34], [35]]. Injections were performed at CT10-11 i.e., at about the start of their active phase. The light pulse experiment was performed by turning on the lights for 1-hr in the room after the two-week dietary intervention in DD (as explained above). A subset of mice was kept in DD as a reference point for the effect of the light pulse.

Tissues were harvested immediately after cervical dislocation and snap-frozen in liquid nitrogen. To harvest serum, blood samples were placed on ice for 30 min followed by centrifugation (3000 rpm, 10 min, 4 °C). Samples were stored at -80 °C prior to downstream analyses.

### 2.3 Tryptophan flux procedure

Mice were acclimated to 12hr light/dark cycles or constant darkness for one week. On the seventh day, U-[13C]-Tryptophan was injected, intraperitoneally at ZT/CT4 or ZT/CT16. Mice were euthanized 5 min and 20 min after injections.

Sample preparation and metabolite extraction: For tissue harvest, mice were euthanized via cervical dislocation and tissues were immediately collected and snap-frozen in liquid nitrogen with a pre-cooled Wollenberger clamp. For serum, blood samples were placed on ice for 30 min followed by centrifugation (3000 rpm, 10 min, 4 °C). Serum (5 μL) was mixed with 150 μL -20 °C 40:40:20 methanol: acetonitrile: water (extraction solvent), vortexed, and immediately centrifuged at 16,000×*g* for 10 min at 4 °C. The supernatant (100 μL) was collected for LC-MS analysis. Frozen tissue samples were ground at liquid nitrogen temperature with a Cryomill (Retsch, Newtown, PA). To minimize data variation due to tissue heterogeneity, entire tissues were harvested and grounded. The resulting tissue powder was weighed and then extracted by adding -20 °C extraction solvent (as above), vortexed, and centrifuged at 16,000×*g* for 10 min at 4 °C. The volume of the extraction solution (μL) was 40x the weight of tissue (mg) to make an extract of 25 mg tissue per mL solvent. The supernatant (40 μL) was collected for LC-MS analysis.

*LC-MS*: A quadrupole-orbitrap mass spectrometer (Q Exactive, Thermo Fisher Scientific, San Jose, CA) operating in a positive ion mode was coupled to vanquish UHPLC system (ThermoFisher Scientific, San Jose, CA) with electrospray ionization and used to scan from *m*/*z* 70 to 1000 at 1 Hz and 140,000 resolutions. The LC separation was achieved on an XBridge BEH Amide column (2.1 mm × 150 mm, 2.5 μm particle size, 130 Å pore size; Waters, Milford, MA) using a gradient of solvent A (95:5 water: acetonitrile with 20 mM ammonium acetate and 20 mM ammonium hydroxide, pH 9.45) and solvent B (acetonitrile). The flow rate was 150 μL/min. The LC gradient was: 0 min, 85% B; 2 min, 85% B; 3 min, 80% B; 5 min, 80% B; 6 min, 75% B; 7 min, 75% B; 8 min, 70% B; 9 min, 70% B; 10 min, 50% B; 12 min, 50% B; 13 min, 25% B; 16 min, 25% B; 18 min, 0% B; 23 min, 0% B; 24 min, 85% B; 30 min, 85% B. Autosampler temperature was 5 °C, and injection volume was 3 μL. Data were analyzed using the MAVEN software. Isotope labeling was corrected for natural 13C abundance using the AccuCor R code [36].

### 2.4 Locomotor activity analysis

Mice were individually housed in cages equipped with an optical beam motion detector (Philips Respironics) throughout the interventions. Data was collected using the Minimitter VitalView 5.0 data acquisition software. Analysis and actograms were performed and generated using Clocklab within the Matlab software (Actimetrics).

### 2.5 Indirect calorimetry

Indirect calorimetry was carried out by negative-flow system cages Oxymax/CLAMS (Columbus Instruments). The mice were placed in the cages on the 11th day of the dietary intervention and metabolic measurements were collected over 72-h under 12-h light/dark cycles or in constant darkness. The first 24-h was an acclimation period and therefore excluded from the analysis.

### 2.6 Food intake, body weight and body composition measurements

Initial food allocation was measured and then remeasured every other day at ∼ZT/CT11. Mice fed *ad libitum*. Likewise, initial body weights were measured at day 0 of the diet interventions and every other day thereafter for the entirety of the intervention. Body composition measurements were taken using EchoMRI™ Whole Body Composition Analyzer on the 14th day of the diet intervention.

### 2.7 Metabolomics analyses

Samples were extracted with methanol and subjected to non-targeted MS analysis using UPLC-MS/MS, as described [37]. Briefly, all methods utilized a Waters ACQUITY ultra-performance liquid chromatography (UPLC) and a Thermo Scientific Q-Exactive high resolution/accurate mass spectrometer interfaced with a heated electrospray ionization (HESI-II) source and Orbitrap mass analyzer operated at 35,000 mass resolution. Sample extracts were dried then reconstituted in solvents compatible with each of the four methods. Each reconstitution solvent contained a series of standards at fixed concentrations to ensure injection and chromatographic consistency. One aliquot was analyzed using acidic positive ion conditions, chromatographically optimized for more hydrophilic compounds. In this method, the extract was a gradient eluted from a C18 column (Waters UPLC BEH C18-2.1 × 100 mm, 1.7 μm) using water and methanol, containing 0.05% perfluoropentanoic acid (PFPA) and 0.1% formic acid (FA). Another aliquot was also analyzed using acidic positive ion conditions; however, it was chromatographically optimized for more hydrophobic compounds. In this method, the extract was a gradient eluted from the same aforementioned C18 column using methanol, acetonitrile, water, 0.05% PFPA and 0.01% FA and was operated at an overall higher organic content. Another aliquot will be analyzed using basic negative ion optimized conditions using a separate dedicated C18 column. The basic extract was a gradient eluted from the column using methanol and water, however with 6.5 mM Ammonium Bicarbonate at pH 8. The fourth aliquot was analyzed via negative ionization following elution from a HILIC column (Waters UPLC BEH Amide 2.1 × 150 mm, 1.7 μm) using a gradient consisting of water and acetonitrile with 10 mM Ammonium Formate, pH 10.8. The MS analysis alternated between MS and data-dependent MSn scans using dynamic exclusion. The scan range methods covered 70–1000 *m*/*z*.

Samples were analyzed in longitudinal batches reflecting the temporal collection of the CLSA cohort approximately every 3 years. Within each temporal batch, metabolites were identified by automated comparison of the ion features in the experimental samples to a reference library of chemical standard entries that included retention time, molecular weight (*m*/*z*), preferred adducts, and in-source fragments as well as associated MS/MS spectra and curated by visual inspection for quality control using software developed at Metabolon. Identification of known chemical entities was based on comparison to metabolomic library entries of purified standards. Commercially available purified standard compounds have been acquired for the determination of their detectable characteristics. Additional mass spectral entries have been created for structurally unnamed biochemicals, which have been identified by virtue of their recurrent nature (both chromatographic and mass spectral). Peaks were quantified using area-under-the-curve. The raw area counts for each metabolite in each sample were normalized to correct for variation resulting from instrument inter-day tuning differences by the median value for each instrument run-day, therefore, setting the medians to 1.0 for each run. Missing values were imputed with the observed minimum after normalization. Pathway enrichment of metabolic pathways was calculated using the following formula (k/m)/((n-k)/(N-m)) where k is the number of significant metabolites within the pathway, m is the total number of metabolites within the pathway, n is the total number of significant metabolites in all pathways and N is the total number of detected metabolites in all pathways. Metabolite data were log transformed and auto-scaled before correlation analysis. Pearson's correlation coefficient was calculated to estimate inter- and intra-tissue metabolite pair correlations. Networks are based on metabolite-pair correlations with an estimated p-value < 0.001.

### 2.8 RNA extraction

Total RNA was extracted from the suprachiasmatic nucleus (SCN) and the liver with TRIzol (Invitrogen), followed by precipitation with isopropanol and ethanol.

### 2.9 Real-Time Quantitative PCR

Complementary DNA (cDNA) was obtained by retrotranscription of 500 ng of RNA from SCNs and 1000 ng of RNA from livers with the Maxima First Strand cDNA Synthesis Kit (Thermo Fisher Scientific). Quantitative real-time polymerase chain reaction (qRT-PCR) analysis was performed using QuantStudio 3 (Applied Biosystems) with PowerUp SYBR Green Master Mix (Applied Biosystems). *18S* was used as an endogenous control for liver samples and *Tbp* was used for SCN samples. The sequences of primer used are as follows: mouse *18S*, 5′-CGCCGCTAGAGGTGAAATTC-3′ (forward) and 5′-CGAACCTCCGACTTTCGTTCT-3′ (reverse); mouse *Tbp*, 5′-CCCTTGTACCCTTCACCAAT-3′ (forward) and 5′-TTGAAGCTGCGGTACAATTC-3′ (reverse); mouse *Nr1d1*, 5′-AGGCTGCTCAGTTGGTTGTT-3′ (forward) and 5′-CTCCATCGTTCGCATCAATC-3′ (reverse); mouse *Dbp*, 5′-AATGACCTTTGAACCTGATCCCGCT-3′ (forward) and 5′-GCTCCAGTACTTCTCATCCTTCTGT-3′ (reverse); mouse *Arntl*, 5′-GCAGTGCCACTGACTACCAAGA-3′ (forward) and 5′-TTGCAATCTTACCCCAGACA-3′ (reverse); mouse Clock, 5′-ACCACAGCAACAGCAACAAC-3′ (forward) and 5′-GGCTGCTGAACTGAAGGAAG-3′ (reverse); mouse *Per2*, 5′-CGCCTAGAATCCCTCCTGAGA-3′ (forward) and 5′-CCACCGGCCTGTAGGATCT-3′ (reverse); mouse *Cry1*, 5′-CAGACTCACTCACTCAAGCAAGG-3′ (forward) and 5′-TCAGTTACTGCTCTGCCGCTGGAC-3′ (reverse); mouse *Nampt*, 5′-GGTCATCTCCCGATTGAAGT-3′ (forward) and 5′-TCAATCCAATTGGTAAGCCA-3′ (reverse) and mouse *Timeless*, 5′- AATACCTGAAACGCTTCGC-3′ (forward) and 5′- TTGAAGAGGCAGAACAGGG-3′ (reverse).

### 2.10 RNA-sequencing and analysis

Total RNA was monitored for quality control using the Agilent Bioanalyzer Nano RNA chip and Nanodrop absorbance ratios for 260/280 nm and 260/230 nm. Library construction was performed according to the Illumina TruSeq® Stranded mRNA Sample Preparation Guide. The input quantity for total RNA was 1000 ng for liver samples and 200 ng for SCN samples and mRNA was enriched using oligo dT magnetic beads. The enriched mRNA was chemically fragmented for 3 min. First strand synthesis used random primers and reverse transcriptase to make cDNA. After second strand synthesis, the ds cDNA was cleaned using AMPure XP beads and the cDNA was end-repaired and then the 3’ ends were adenylated. Illumina barcoded adapters were ligated on the ends and the adapter-ligated fragments were enriched by nine cycles of PCR. The resulting libraries were validated by qPCR and sized by Agilent Bioanalyzer DNA high sensitivity chip. The concentrations for the libraries were normalized and then multiplexed together. The multiplexed libraries were sequenced on paired-end 100 cycles chemistry on the Novaseq 6000. The version of Novaseq control software was NVCS ver 1.7.0 with real-time analysis software, RTA 3.4.4.

### 2.11 Bioinformatics and pathway analyses

The reads from each replicate experiment were aligned to the reference genome assembly mm10 and corresponding transcriptome using Tophat. Subsequently, gene expression levels were computed from the read alignment results using Cufflinks, another tool in the Tuxedo protocol. This protocol outputs the FPKM values for each gene of each replicate. To determine the periodicity of genes, the JTK-CYCLE [38] algorithm and the BIO_CYCLE [39] algorithm were used and produced consistent results. The output of these algorithms includes the amplitude, phase, and p-value for each transcript. Genes were considered circadian if their p-value outputted by JTK-CYCLE was <0.05. Heatmaps of circadian transcripts were generated using the R package gplots v3.0.3, where the rows were sorted by the JTK_CYCLE output phase and row z-score normalized. Gene ontology and pathway analysis was performed using DAVID software [40].

### 2.12 Protein extraction and Western blot

Frozen liver pieces were placed in 1.5 ml Eppendorf tubes with RIPA buffer (50 mM Tris-HCl [pH 8.0], 150 mM NaCl, 1% NP-40, 0.5% Sodium Deoxycholate, 0.1% SDS, 5 mM MgCl2, and 1 mM PMSF) supplemented with Protease Inhibitor Cocktail (Roche), 1 mM DTT, 20 mM NaF, 10 mM Nicotinamide, 330 nM Trichostatin A (T8552, Sigma). Samples were homogenized using a VWR 200 homogenizer followed by a 10 s sonication at 60%. The tissue lysates were centrifugated at 13200 rpm for 15 min at 4 °C. Supernatants were transferred to a new tube and an aliquot was used for protein quantification using the Bradford assay (Bio-Rad). A total of 20 μg of protein lysates were separated on 8% gels by SDS-PAGE and transferred to nitrocellulose membranes. The membranes were incubated with primary antibodies overnight (anti-BMAL1, Abcam, ab93806; anti-REVERBα, Cell Signaling Technologies, 13418; anti-CLOCK, Bethyl Labs, A302-618A; anti-CRY1, Bethyl Labs, A302-614A; anti-PER2, Alpha Diagnostic, PER21-A; anti-P84, Genetex, GTX70220) at 4 °C and peroxidase-conjugated secondary antibodies (Anti-mouse IgG, HRP conjugate, EMD Millipore, AP160P; Anti-rabbit IgG, HRP-linked, EMD Millipore, 12-348) for 1 h at room temperature. Visualization was performed by chemiluminescent HRP substrate (WBKLS0500, EMD Millipore). The blots were developed with autoradiography films (HyBlot CL, Denville Scientific). The films were scanned and densitometry was analyzed through ImageJ software.

### 2.13 Statistics

For each experiment, the number of biological replicates, statistical test, significance threshold and visual representation information (i.e., mean, SEM, etc. of graphs) can be found in the figure legends. Power analyses were performed to determine the number of samples using G-power 3.1 analyses software. A power >0.8 was considered sufficient. Complex statistical analyses are described within their corresponding methods section. Unless otherwise stated, data were analyzed in Prism 9.0 (GraphPad).

## 3 Results

### 3.1 Essential amino acids alter circadian rhythms of locomotor activity

**Figure 1:**
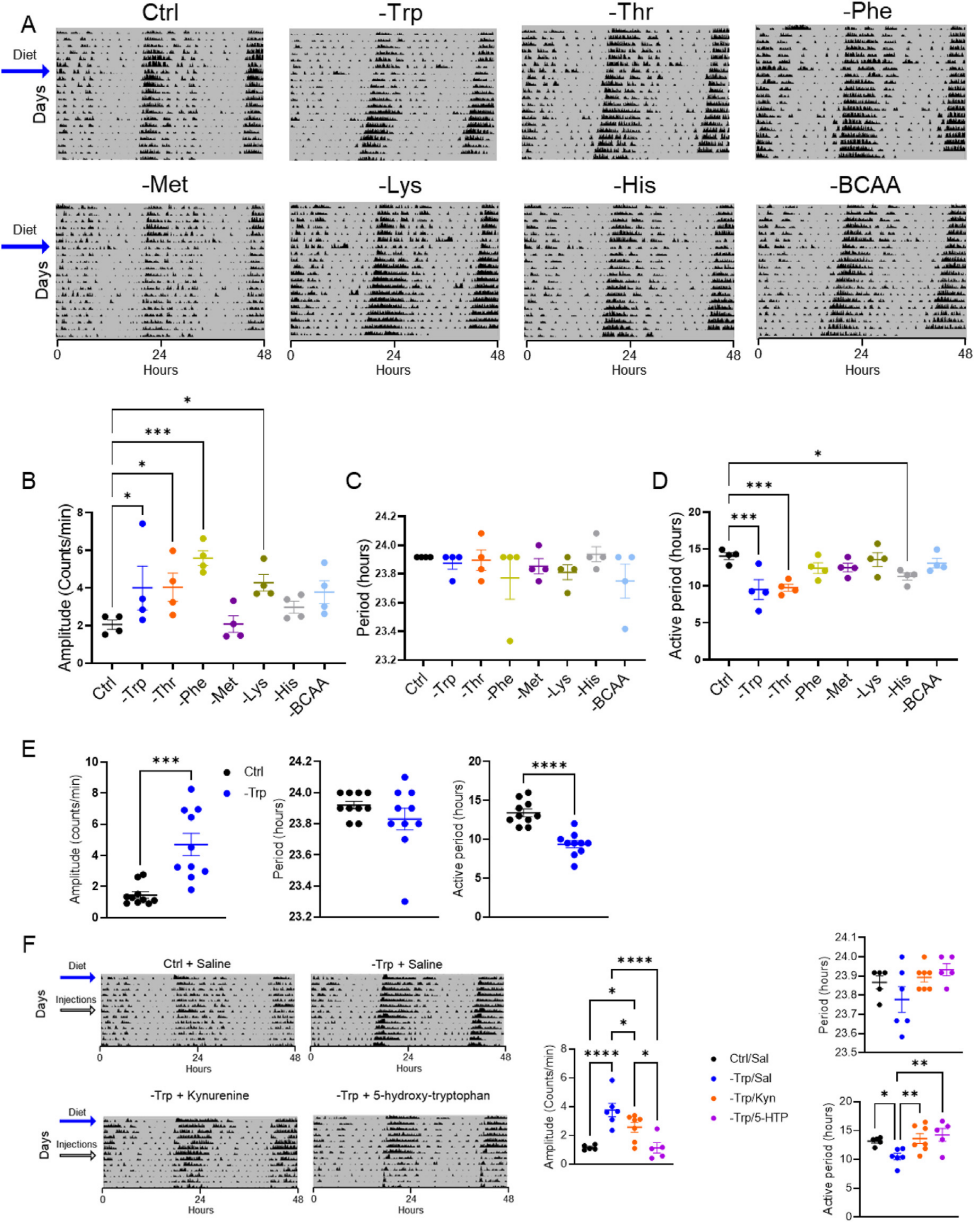
Essential amino acids regulate the circadian locomotor activity. **A**. Actograms representing the locomotor activity of mice fed diets depleted of specific essential amino acids and housed in constant darkness (indicated by the dark background). Black arrows on the left points on the start of the dietary intervention. Mice were fed regular chow until the start of the dietary intervention. **B-D**. Cosinor analyses of the last 72 h of the dietary interventions determined amplitude (**B**), period (**C**), and length of the active period (**D**). N = 4 mice. **E**. Actograms mice fed a chow diet (Ctrl) or a tryptophan depleted diet (-Trp) and the graphs representing the calculated amplitude, period and the length of the active period. N = 10 mice. **F.** Actograms showing the locomotor activity of mice injected with saline or the indicated tryptophan metabolites as well as cosinor analyses of the last 72 h of the dietary interventions. Unpaired student's t-test was used to determine significant differences between the two groups. One-way ANOVA was performed to determine statistically significant differences between more than two groups and multiple comparisons were subsequently analyzed with Fischer's least significant difference test. ∗*P* < 0.05, ∗∗*P* < 0.01, ∗∗∗*P* < 0.001 and ∗∗∗∗*P* < 0.0001. Data are mean ± s.e.

To gain insight into the role of EAAs in regulating central circadian rhythms, we fed C57Bl6/J mice diets depleted of each EAA for 2 weeks under constant darkness (DD) to avoid overriding effects of photic *zeitgeber*-signals. For branched-chain amino acids, we depleted all three together because their catabolism overlap. Most of EAAs influenced behavioral rhythms of locomotion when they are depleted in diet (Figure 1A). Cosinor analyses of the last 72 h of the intervention revealed that the amplitude of locomotor activity was significantly increased in mice fed diets depleted of tryptophan, threonine, phenylalanine or lysine (Figure 1B). While there were no significant changes in period (time between bouts of activity) (Figure 1C), the length of the activity bout (the time mice were active in each bout) was significantly shorter in mice fed diets depleted of tryptophan, threonine, or histidine (Figure 1D). Thus, only tryptophan or threonine depletion altered both the amplitude and the activity period. We repeated the experiment in a larger cohort which confirmed the effect of tryptophan depletion on locomotor activity (Figure 1E). Based on these data and previously known effects of tryptophan-derived metabolites (e.g., serotonin, kynurenine, and NAD^+^) on circadian homeostasis, we decided to focus on the role of tryptophan (Trp) metabolism in regulating circadian rhythm.

We next asked whether tryptophan metabolites can reverse the effects mediated by Trp-depletion. Thus, mice were subjected to the dietary intervention as described above for one week and were injected with saline, kynurenine or 5-hydroxytryptophan (5-HTP) at CT10-11 daily throughout the second week of the intervention. Kynurenine partially rescued the phenotype while 5-HTP fully reversed the -Trp induced effects (Figure 1F), suggesting that effects are not induced by general amino acid deficiency.

### 3.2 Tryptophan metabolism is regulated by environmental cues

**Figure 2:**
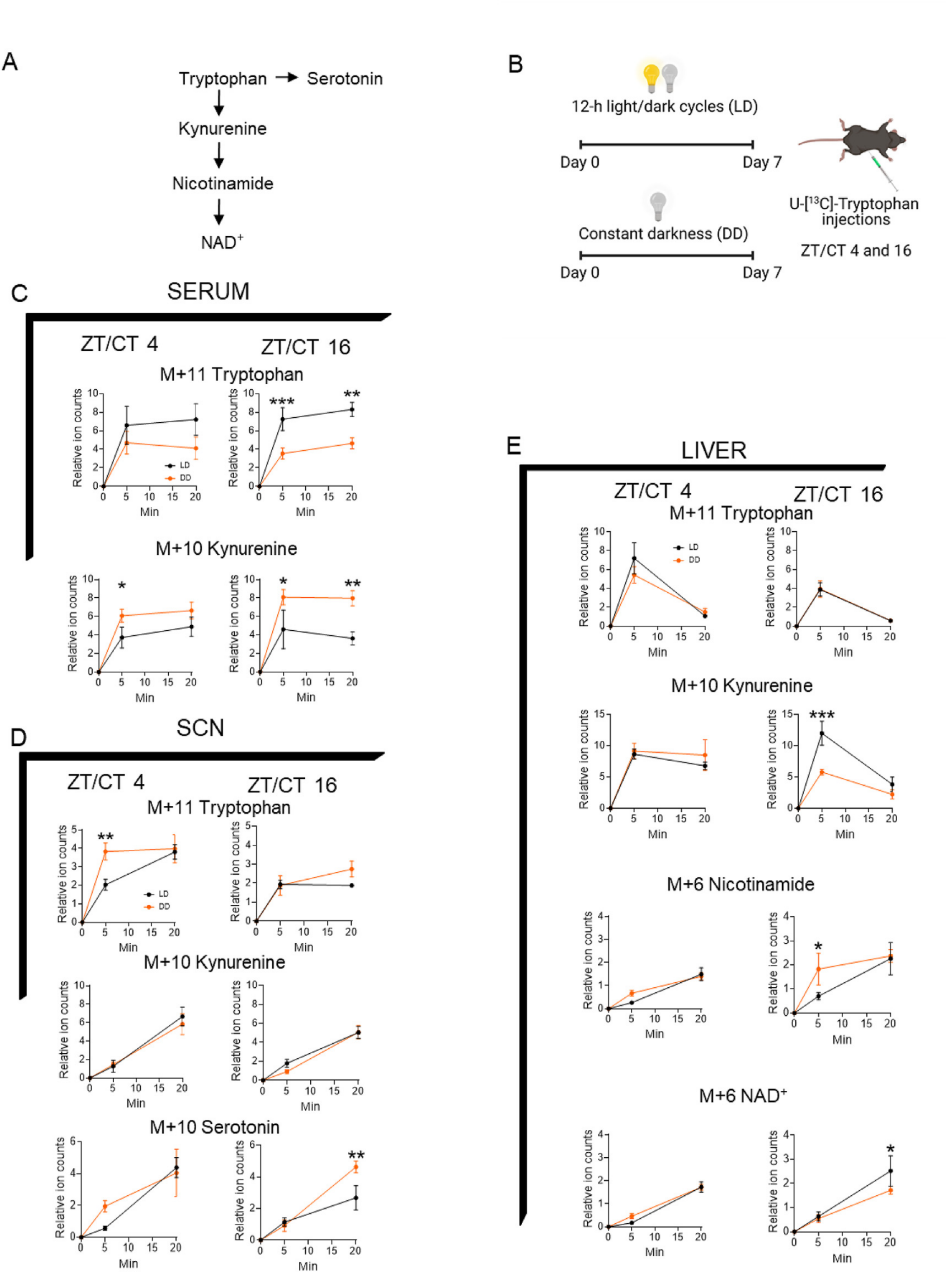
Tryptophan metabolism is time and light-sensitive. **A**. Schematic overview of the tryptophan pathway. **B**. Schematic representation of the tryptophan isotope tracing. **C-E**. Graphs show labeled ion counts of the indicated metabolites in the serum (**C**), SCN (**D**) and liver (**E**). N = 5 mice. Two-way ANOVAs followed by Fischer's least significant difference tests were used to determine significant differences. ∗*P* < 0.05 and ∗∗*P* < 0.01. Data are mean ± s.e.

The effects of Trp metabolism on behavioral rhythms led us to ask whether Trp catabolism is time and/or light-dependent. It is noteworthy that melatonin, a Trp-derived hormone that is suppressed by light and regulates circadian homeostasis [41], is not produced in C57Bl6/J mice [42,43]. However, they produce other key circadian rhythm-related metabolites such as serotonin [[44], [45], [46]], kynurenine [47] and NAD^+^ [48,49], rendering these mice as an appropriate strain for studying the melatonin-independent effects of Trp on circadian physiology. Thus, we first tested whether these metabolites are generated from Trp in a time- and/or light-dependent manner (Figure 2A). To this end, we performed *in vivo* Trp isotope tracing experiments under light-dark (LD) or constant dark (DD) for 1 week. Based on the phase of the locomotor activity (Figure 1), we chose *zeitgeber* time/circadian time 4 (ZT/CT4) and ZT/CT16 (Figure 2B). Serum, liver and the SCN were collected 5 min and 20 min after [U-^13^C]-Trp provision and Trp-derived labeled metabolites were measured by liquid chromatography-mass spectrometry (LC-MS). In serum, both labeled Trp and kynurenine reached steady-state at 5 min, indicating rapid systemic Trp metabolism (Figure 2C). Constant darkness caused higher production of kynurenine despite lower labeled Trp levels. This DD-induced accelerated kynurenine production was more substantial at ZT/CT16 compared to that at ZT/CT4.

The SCN showed slower Trp metabolism, with the levels of labeled downstream metabolites increased over time (Figure 2D). While the kynurenine production rate was indistinguishable among different conditions, serotonin production was accelerated by DD at ZT/CT16. These data suggest the role of light/dark cycles in regulating central serotonin production. Compared to the SCN, liver Trp metabolism was faster, showing peak levels of labeled Trp and kynurenine at 5 min (Figure 2E). The production of further downstream metabolites such as nicotinamide and NAD^+^ was slower. Notably, at ZT/CT16, DD showed lower labeled kynurenine but higher labeled nicotinamide, suggesting accelerated Trp metabolism. This was consistent with the higher labeled circulating kynurenine (Figure 2C), which is mostly produced from the liver [50]. Thus, light and time govern systemic Trp metabolism.

To gain further insight on the inter-relationship between Trp metabolism, circadian rhythms and environmental cues, we revisited our metabolomics datasets of high-fat diet-induced obesity [22] and physical exercise [51], both of which rewired circadian metabolic homeostasis. High-fat diet feeding altered the levels and rhythms of Trp metabolites in the liver, serum and SCN (Figures S1A–C). In particular, the rhythm of kynurenine in the liver and serum was dampened (Figures S1A and B). Physical exercise also altered Trp metabolism in a time-dependent manner, with kynurenine systemically induced in mice exercising at ZT16 (Figures S1D–F). Together, circadian regulation of Trp metabolism is sensitive to various interventions such as light exposure, diet and exercise, supporting its role as a physiological integrator of environmental cues.

### 3.3 Tryptophan and light coordinate temporal regulation of systemic metabolism

We next investigated the circadian (in constant darkness) and diurnal (in the presence of light/dark cycles) effects of dietary Trp depletion on other metabolic parameters. To this end, we exposed mice to LD and DD for one week before feeding a diet lacking Trp (-Trp) or a control diet (Ctrl) while keeping the same light schedule for an additional two weeks (Figures S2A and B). The two-week period was determined based on our intervention in Figure 1 and on previous data demonstrating that dietary Trp depletion accelerated weight loss starting at around day 13 [52]. Consistently, we also observed that -Trp fed mice lost a significant amount of body weight in both LD and DD which resulted in leaner body composition (Figures S2C and D). Interestingly, the body weight loss was not due to reduced food intake (Figures S2E and F) but likely due to increased energy expenditures (Figures S2G–H).

**Figure 3:**
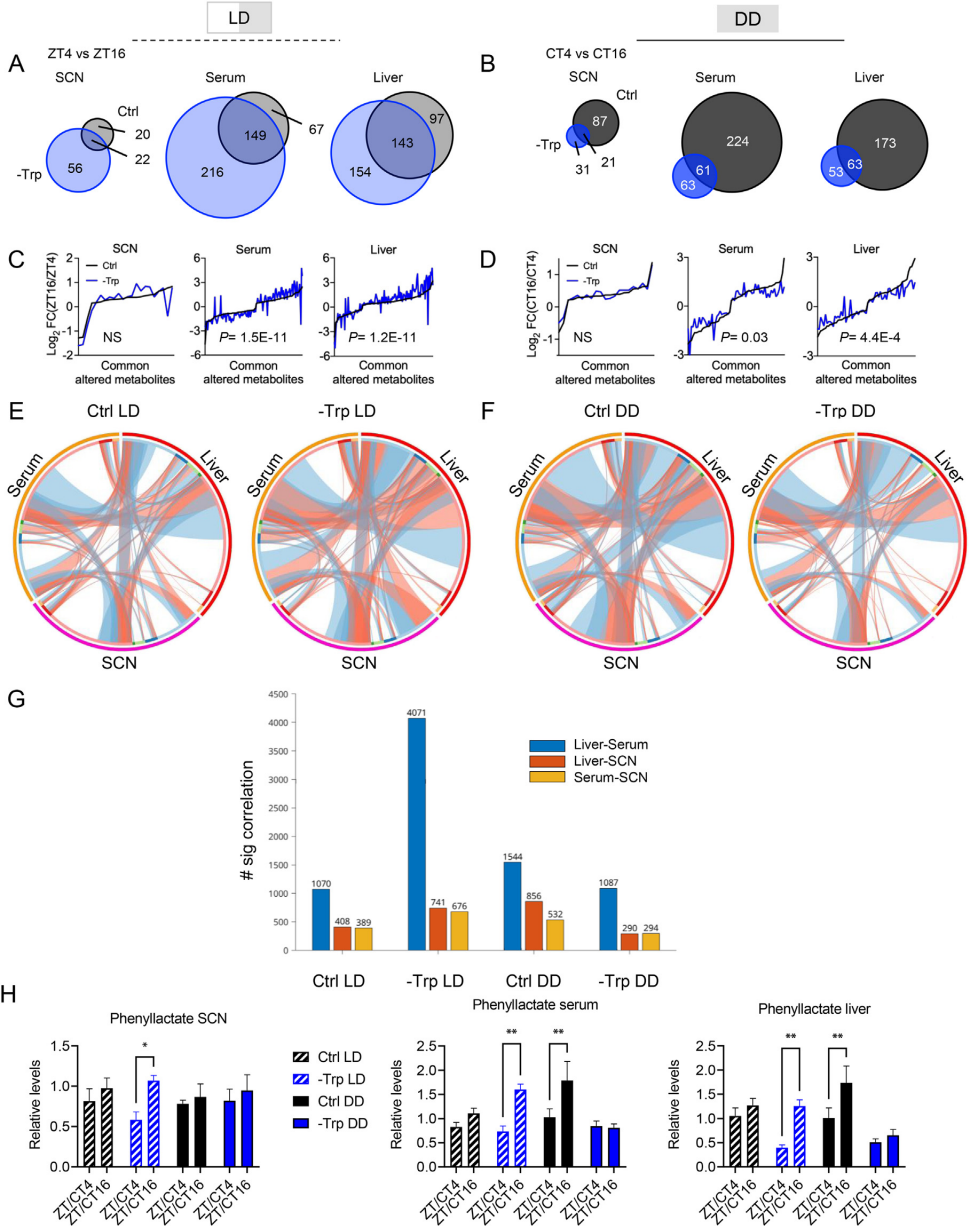
Temporal regulation of systemic metabolism is coordinated by light and tryptophan. **A-B**. Venn diagram representing the overlap of temporally regulated (comparing ZT4 and 16) metabolites between control (Ctrl) and tryptophan depletion (-Trp) in the SCN, serum and the liver. Mice were housed under 12-h light/dark cycles (**A**) or constant darkness (**B**). **C-D**. Log_2_ fold change (ZT16/ZT4) of the overlapping metabolites under 12-h light/dark cycles (**C**) or constant darkness (**D**). **E-F**. Circos plots represent correlations of metabolites between tissues from mice housed under light/dark cycles (**E**) or constant darkness (**F**). Red connections represent positive correlations and blue connections represent negative correlations. **G**. Bar chart showing the number of significantly correlating metabolites between the tissues and the serum in each group. **H.** Phenyllactate levels in the SCN, serum and liver at two time points illustrated as bar charts. Significant differences between time points were determined using a Two-Way ANOVA. Significant differences in the temporal fold change between dietary interventions were determined by first converting all negative values to positive followed by a paired students t-test of the Log_2_ fold changes and Bonferroni correction. N = 5 mice. NS = not significant. ∗*P* < 0.05 and ∗∗*P* < 0.01. Data are mean ± s.e.

To gain more detailed insight into the temporal regulation of metabolism, we performed metabolomics analyses of the SCN, serum, and liver of these four intervention groups (Table S1). Strikingly, the -Trp diet induced a much larger number of metabolites fluctuating between ZT4 and ZT16 than the Ctrl diet in LD conditions (blue versus gray in Figure 3A). This pattern was observed across the SCN, serum, and liver. In contrast, such response was severely blunted in the absence of a light-dark cycle (dark blue versus black in Figure 3B). Intriguingly, these metabolites whose temporal regulation was sensitive to light and/or dietary Trp were not restricted to the Trp pathway (Figures S3A and B) but included a broad spectrum of central metabolic pathways (Figures S4A–C). Moreover, the overlapping metabolites that were temporally regulated by both diets displayed a significantly higher fold change in LD as compared to DD (Figure 3C,D). The number of metabolites that showed a significant correlation between organs, which reflects inter-organ metabolic coordination, was substantially increased in mice fed a -Trp diet in LD but reduced in DD (Figure 3E–G). Among the metabolites displaying this pattern we identified phenyllactate (Figure 3H) which recently was shown to be an exercise-induced metabolite regulating appetite [53]. Noteworthy is that the -Trp group displayed slightly larger amplitude in eating behavior in LD but not in DD (compare Figures S2G and H). Yet, the feeding pattern was not lost in the -Trp DD group suggesting other factors are at play contributing to the loss of temporal metabolic regulation. Together, dietary tryptophan and light/dark cycles coordinate temporal regulation of metabolism.

### 3.4 Tryptophan contributes to the control of the circadian transcriptome

**Figure 4:**
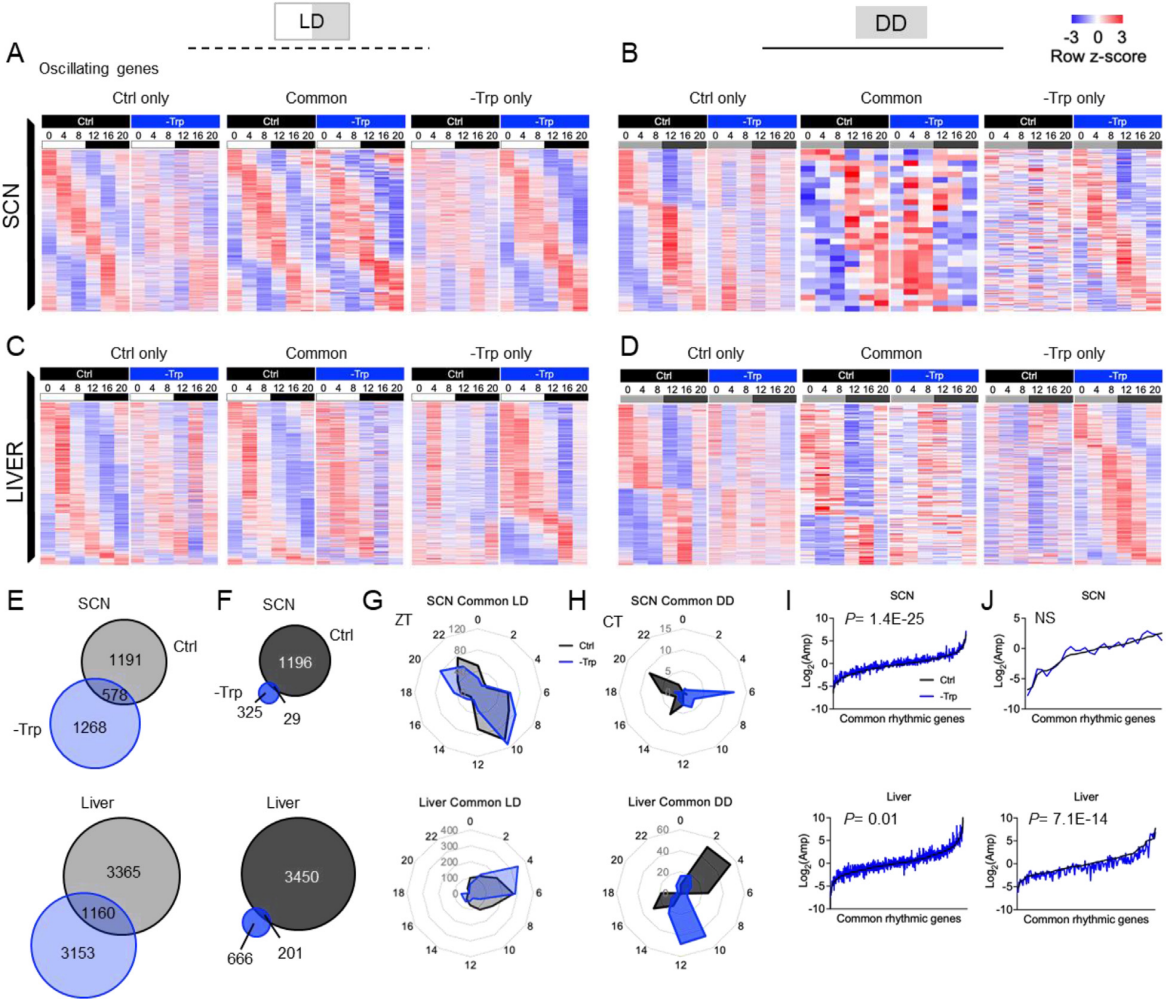
Light and tryptophan coordinate circadian transcriptional rhythms. **A-D**. Heatmaps represent the oscillating genes in the SCN (**A, B**) and the liver (**C, D**) that are common or unique for the dietary groups of mice housed under light/dark cycles (**A, C**) or constant darkness (**B, D**). **E-F**. Venn diagrams represent the overlap of oscillating genes between the dietary intervention groups in the SCN and the liver in mice housed under light/dark cycles (**E**) or constant darkness (**F**). **G-H**. Radar plots represent the phase of the oscillating metabolites of the SCN and liver in mice housed under light/dark cycles (**G**) or constant darkness (**H**). **I-J**. Amplitude for each gene oscillating in both dietary groups under light/dark conditions (**I**) or constant darkness (**J**) plotted in ascending order based on the amplitude in the control groups. Significant oscillations were determined by JTK-CYCLE *P* < 0.05. The amplitudes were statistically compared between dietary groups by a paired students t-test followed by Bonferroni correction. N = 3 mice. NS = not significant.

Metabolism and the core clock system are closely coupled [54,55]. Therefore, we next focused on circadian transcriptional rhythms. Hence, we performed RNA-sequencing analyses on SCNs and livers from mice fed a Ctrl or -Trp diet and housed under LD or DD conditions (Tables S2–3). Tissues were collected at six time points over the circadian cycle and transcriptional rhythms were determined using JTK_cycle [38]. Transcriptional oscillations were observed in both tissues from mice in all four intervention groups (Figure 4A–D). In LD cycles, the rhythmic transcriptional program was largely altered in the absence of dietary Trp, yet transcriptional rhythms were still present (Figure 4E, S5A and B). Conversely, in DD, while several genes oscillated in the control diet group, it was dramatically reduced in -Trp fed mice (Figure 4F, S5C and D), further supporting that dietary Trp is important in sustaining circadian rhythms. Furthermore, in contrast to the well-aligned phases of the oscillating genes between the dietary groups in LD (Figures 4G and S5E), such phases were markedly misaligned in DD (Figure 4H and S5F). Similar to the metabolomics data, commonly oscillating genes between the dietary groups in LD displayed a significantly higher amplitude for the -Trp group (Figure 4I), which was attenuated under constant darkness (Figure 4J). Overall, these metabolomics and transcriptomics data indicate the importance of dietary Trp in buffering photic cues (i.e., dampening the number and amplitude of temporally regulated transcripts and metabolites in LD and sustaining them in DD) to control circadian homeostasis.

### 3.5 Tryptophan metabolites contribute to the control of central and hepatic circadian rhythms

**Figure 5:**
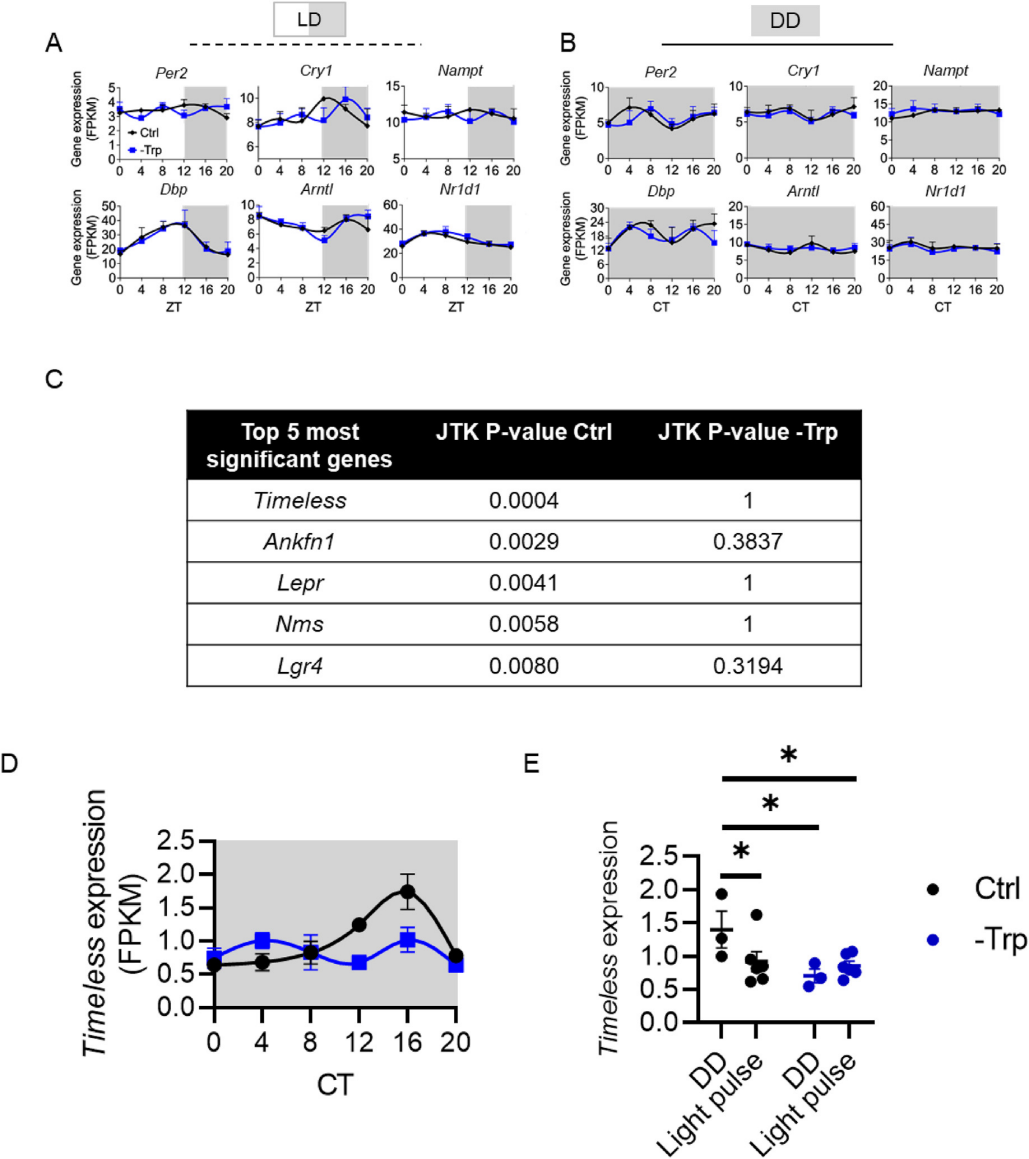
Tryptophan and light regulate *Timeless* expression in the suprachiasmatic nucleus. **A-B**. Gene expression of core clock genes in the SCN of mice housed under light/dark cycles (**A**) or constant darkness (**B**). **C**. Table of the top 5 genes rhythmic Ctrl but not -Trp in DD. **D**. Gene expression of *Timeless* in the SCN in constant darkness over the circadian 24-hr period. **E**. *Timeless* expression in the SCN at CT17 of mice housed in constant darkness (DD) or ZT1 in mice exposed to a 1-hr light pulse at CT16 after being housed in constant darkness for three weeks and fed a tryptophan depleted diet (-Trp) for the two last weeks and compared to mice fed nitrogen matched chow (Ctrl). Statistics were analyzed by two-way ANOVA and subsequently tested with Fischer's least significant difference test. ∗*P* < 0.05. Data are mean ± s.e. N = 3 for mice housed in DD and N = 6 for mice exposed to a light pulse.

We hypothesized that the core clock machinery within the SCN would display induced rhythmicity in LD and dampened rhythms in DD. However, we found no changes in the oscillation of selected circadian genes in both LD and DD (Figure 5A–B). This observation led us to investigate the circadian SCN genes more broadly by using the SCN RNA-seq dataset (Table S2). Filtering out the genes annotated in the ontology pathway “circadian rhythm” (GO:0007623) revealed *Timeless* as the top candidate that only oscillated in the Ctrl DD group (Figure 5C–D). The role of *Timeless* in responding to photic cues was tested by exposing mice to a 1-hr light pulse at CT16 at the end of the dietary intervention. *Timeless* expression was attenuated in the SCN of mice exposed to a light pulse only when fed a Ctrl diet (Figure 5E) suggesting that light and dietary Trp coordinate *Timeless* expression in the SCN. *Timeless* is required for normal circadian rhythms in mammals [56] yet, its role in mediating the -Trp effects on circadian rhythms remains to be elucidated.

**Figure 6:**
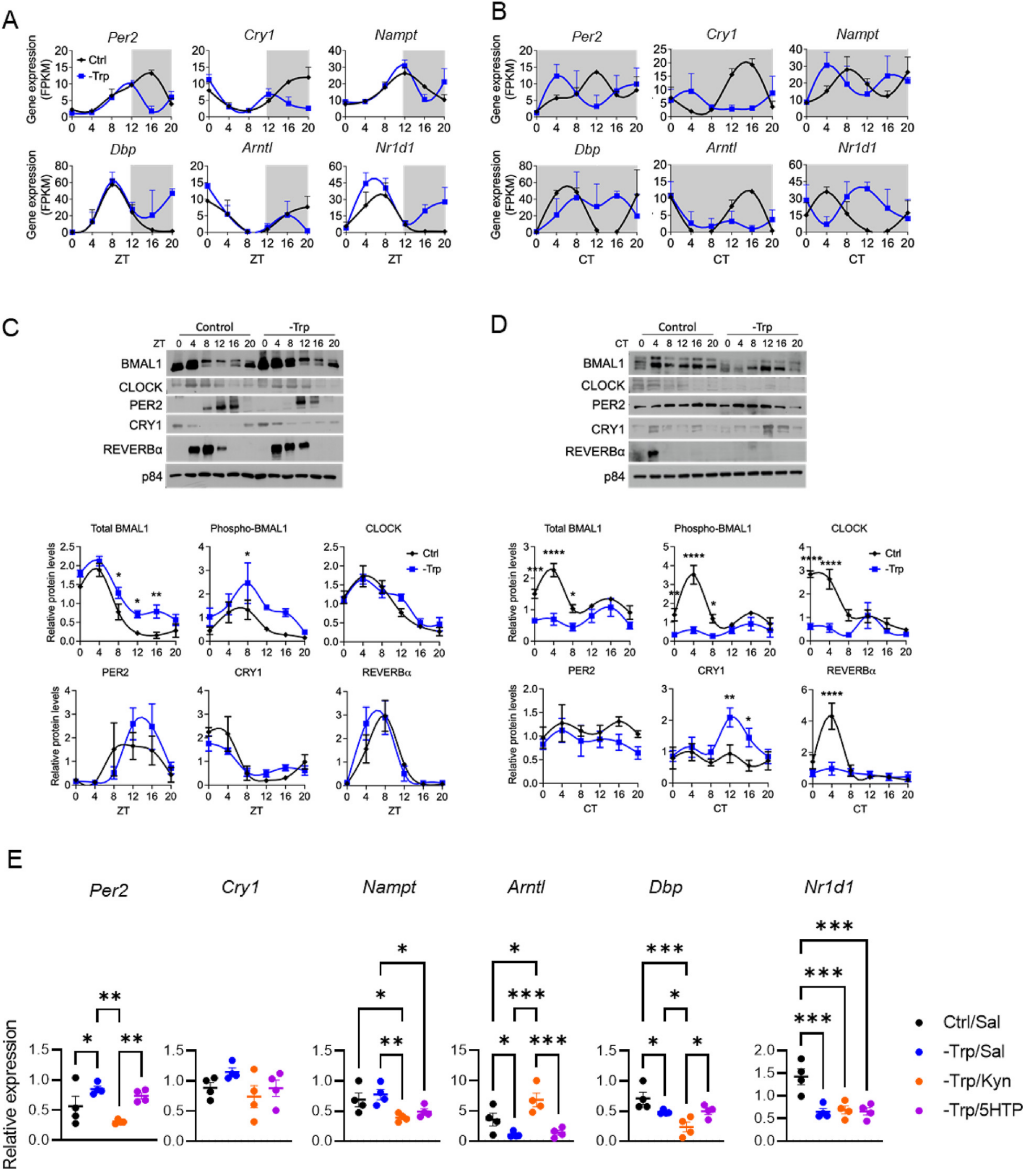
Dietary tryptophan controls the hepatic core clock machinery in a light-dependent manner. **A-B**. Gene expression of core clock genes in the liver of mice housed under light/dark cycles (**A**) or constant darkness (**B**). **C**. Representative western blots of hepatic core clock proteins. The western blot analyses were repeated in n = 4 and the quantification is illustrated in the graphs. **D**. Gene expression of selected circadian genes from mice from the experiment in Figure 1F. Livers were harvested at CT4 after the last injection (CT10-11 the day before). One-way ANOVA was used to determine statistically significant differences between more than two groups and multiple comparisons were subsequently analyzed with Fischer's least significant difference test. ∗*P* < 0.05, ∗∗*P* < 0.01 and ∗∗∗*P* < 0.001. Data are mean ± s.e.

Given that systemic tryptophan metabolism is most active in the liver and that the liver clock is responsive to dietary cues [19], we also investigated the impact of Trp deficiency on the hepatic core clock machinery. Transcriptional rhythms of representative circadian genes were specifically altered during the dark phase in -Trp fed mice under LD (Figure 6A). More importantly, DD caused a loss of oscillation of these key circadian genes in -Trp fed mice (Figure 6B). These findings were supported by protein expression of the clock machinery (Figure 6C–D). Interestingly, kynurenine injections rescued the expression of core clock genes such as *Per2* and *Arntl* while 5-HTP did not rescue any (Figure 6E), indicating mechanisms that are independent of the central clock as 5-HTP had more potent effects on behavioral rhythms (Figure 1F). Hence, our data suggest that Trp metabolism controls circadian homeostasis and that the kynurenine branch may serve as a *zeitgeber* in the liver.

## 4 Discussion

Mammalian circadian homeostasis is regulated on several levels ranging from single cells to tissue specialized inter-organ communication [17,57,58]. Herein, we demonstrate that Trp metabolism is a time- and light-sensitive pathway integrating the photic response to modulate circadian homeostasis. Using multi-omics approaches, we also define the circadian components involved in commonly responding to photic cues and Trp metabolites. Collectively, our study explores the regulation of circadian homeostasis by elucidating the intersection between light and diet.

The link between Trp metabolism and light has been known for decades [[59], [60], [61]]. However, their cooperative effects on physiology and circadian rhythms have not been investigated thoroughly. Our study characterized the physiological coordination between Trp metabolism and photic *zeitgeber* signals. The metabolomics and transcriptomics data concordantly suggest that dietary Trp intake is required for the vast majority of circadian rhythms (free-running rhythms in DD). In contrast, metabolic and transcriptional diurnal rhythms (biological rhythms in LD) are to a certain degree enhanced when dietary Trp is depleted, suggesting that Trp metabolism is buffering photic cues. The metabolic effects of Trp depletion could possibly be explained by the altered expression of circadian genes in the liver [62] and the behavioral effects could be regulated by lost *Timeless* rhythms [56]. This could be tested by providing -Trp to liver-specific *Bmal1* knock-out mice and SCN-specific *Timeless* knock-out mice. However, we do not expect the Trp effects on circadian homeostasis to be explained solely by these genes given the number of physiological processes Trp is involved in. While we did not directly dissect the molecular mechanisms by which Trp metabolism is regulating circadian homeostasis, previous studies have identified multiple potential underlying mechanisms, including metabolites of the kynurenine pathway acting as UV-protectant in the eye [59], the photo-protectant role of serotonergic afferents near and/or on retinal axon terminals in the SCN [44], or NMDAR which binds kynurenine metabolites to regulate behavioral responses to photic signals [63]. Another possibility is that the depletion of Trp resulted in replacement with phenylalanine during translation which subsequently alter the function of the proteins [64].Light can also enhance Trp catabolism, which yields photoproducts that may regulate the expression of circadian genes [65]. Trp photoproducts are aryl hydrocarbon receptor (AhR) agonists which regulate the expression of the core clock machinery and can form a heterodimer with Bmal1 [66]. We did not observe altered expression of the canonical AhR target-genes in our transcriptomics data however, Trp metabolism might influence AhR activity at specific sites on the chromatin. Such mechanisms would suggest that light could synchronize peripheral circadian rhythms independent of the central clock, an intriguing concept suggested by previous work from our lab [67]. Thus, Trp is likely regulating systemic circadian rhythms via multiple molecular mechanisms.

Characterizing Trp metabolism as a fundamental integrator of circadian homeostasis helps elucidate mechanisms linking environmental and behavioral factors to biological rhythms. For instance, in conditions such as fasting, free fatty acids compete with Trp for albumin binding resulting in more free Trp available for tissue uptake [68]. Thus, feeding-fasting cycles not only regulate Trp levels via dietary delivery but also the availability for uptake in the circulation, which may contribute to the link between the clock and time-restricted feeding [2,18,19]. Furthermore, physical exercise regulates kynurenine metabolism in the muscle by limiting its availability for uptake to the brain [47]. It is tempting to speculate that a trained muscle limits the availability of kynurenine to the brain and hence allows for a more potent synchronization by light in the SCN, a possible mechanism linking physical exercise and circadian rhythms [69,70]. The *Ad hoc* analyses of our published work [22,51] suggest that oscillations of kynurenine are dampened upon high-fat diet feeding and strongly induced after exercise at ZT15 specifically. This observation, in combination with our data, suggests that physical exercise could rescue circadian rhythmicity in individuals with obesity via the regulation of systemic kynurenine oscillations. In fact, the notion that Trp metabolites are mediating their physiological effects by integrating behavioral signals are further supported by findings that Trp supplementation is deleterious for health in sedentary mice while it is beneficial in exercising mice [71]. Furthermore, we found that phenyllactate is temporally regulated in a tryptophan- and light-dependent manner which might contribute to the metabolic and behavioral phenotype observed in these mice [53]. However, tryptophan depletion altered metabolic pathways in a tissue-specific manner, suggesting that different metabolites likely contribute to the Trp-mediated effects in the liver and SCN. Our systems biology approach sets the stage for functional studies focusing on the tissue-specific mechanisms contribuiting to the control of circadian physiology. This likely involves neuroactive and immunometabolic processes regulated by Trp derivatives [72].

A limitation of this study is the lack of full circadian/diurnal analyses for some assays such as the metabolomics data. For instance, we cannot distinguish between altered amplitudes and phase shifts based on data from two time points. However, the effect on the metabolome is corroborated by the transcriptome data that where we had six time points.

In conclusion, our findings introduce Trp metabolism as a circadian modulator that moderates diurnal rhythms and sustains circadian rhythms. Trp metabolism has been suggested as an attractive drug target for different diseases such as cancers and neurodegeneration [72] and the work presented here may indicate Trp-mediated circadian biology as underlying physiology for such therapeutic interventions [7,73].

## Author contributions

P.P., M.C., P.B., P.K. and P.S.-C. designed the study and analyzed the data. P.P., M.C., T.S., S.S., K.B.K., G.P., Y.A. and N.M. performed experiments and/or interpreted the results. A.C. conducted the tryptophan isotope tracing under the guidance of C.J. M.S. and P.B. performed the bioinformatics analyses. M.M.S. performed the cross-tissue gene correlation analysis. J.M.M.K., K.A.D. and D.L. conducted global metabolite and transcriptomic profiling. P.P., M.C., and C.J. wrote the paper with feedback from all authors.

## Data Availability

The RNA-seq data are available through the gene omni bus portal (GSE186242 for the SCN and GSE190145 for the liver) as well as from the CircadiOmics web portal at: circadiomics.ics.uci.edu [74,75].
