# Supplementary figures and images for: Tryptophan metabolism is a physiological integrator regulating circadian rhythms
Source: Mol Metab. 2022 Jul 29;64:101556. doi: 10.1016/j.molmet.2022.101556 (PMC9382333; doi:10.1016/j.molmet.2022.101556)

## Slide 1
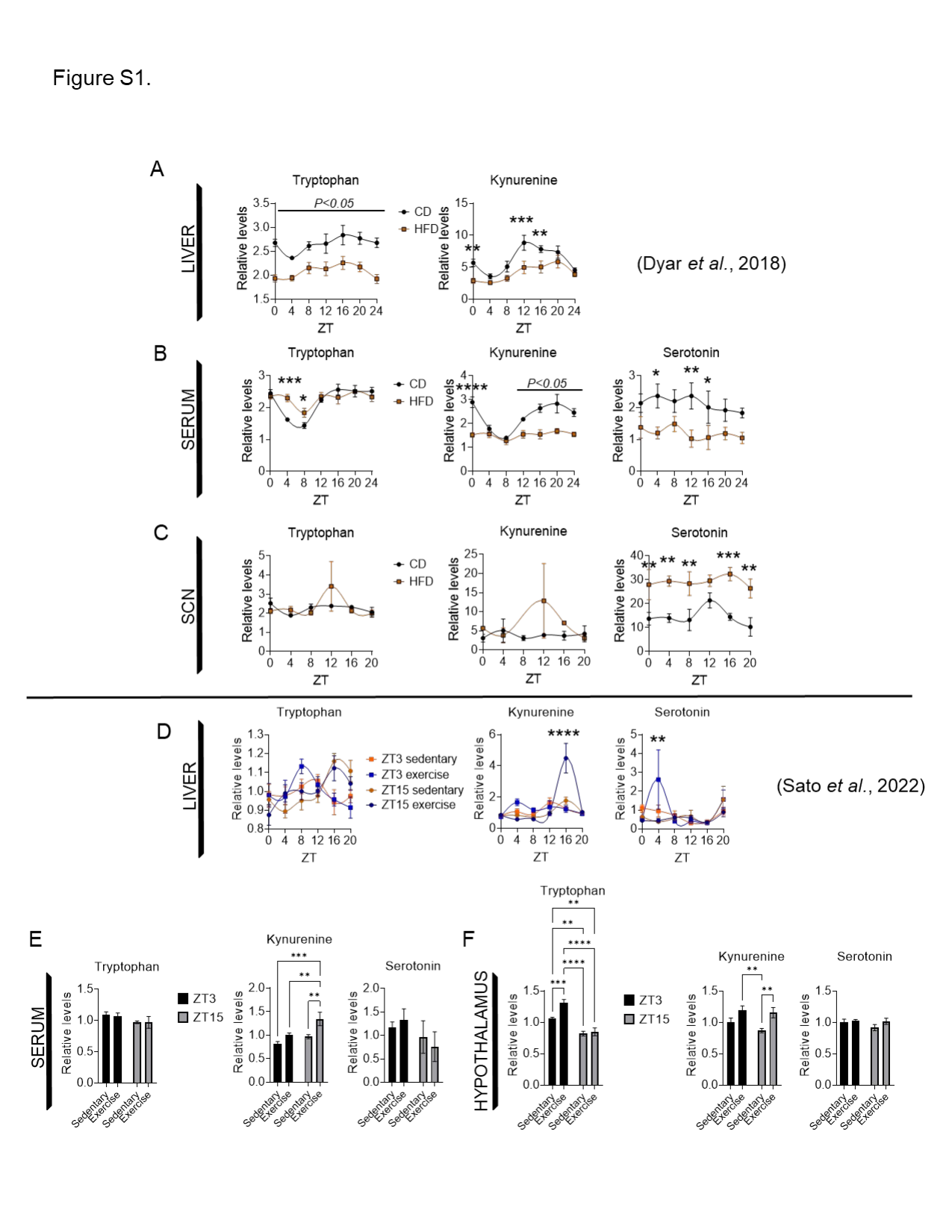

## Slide 2
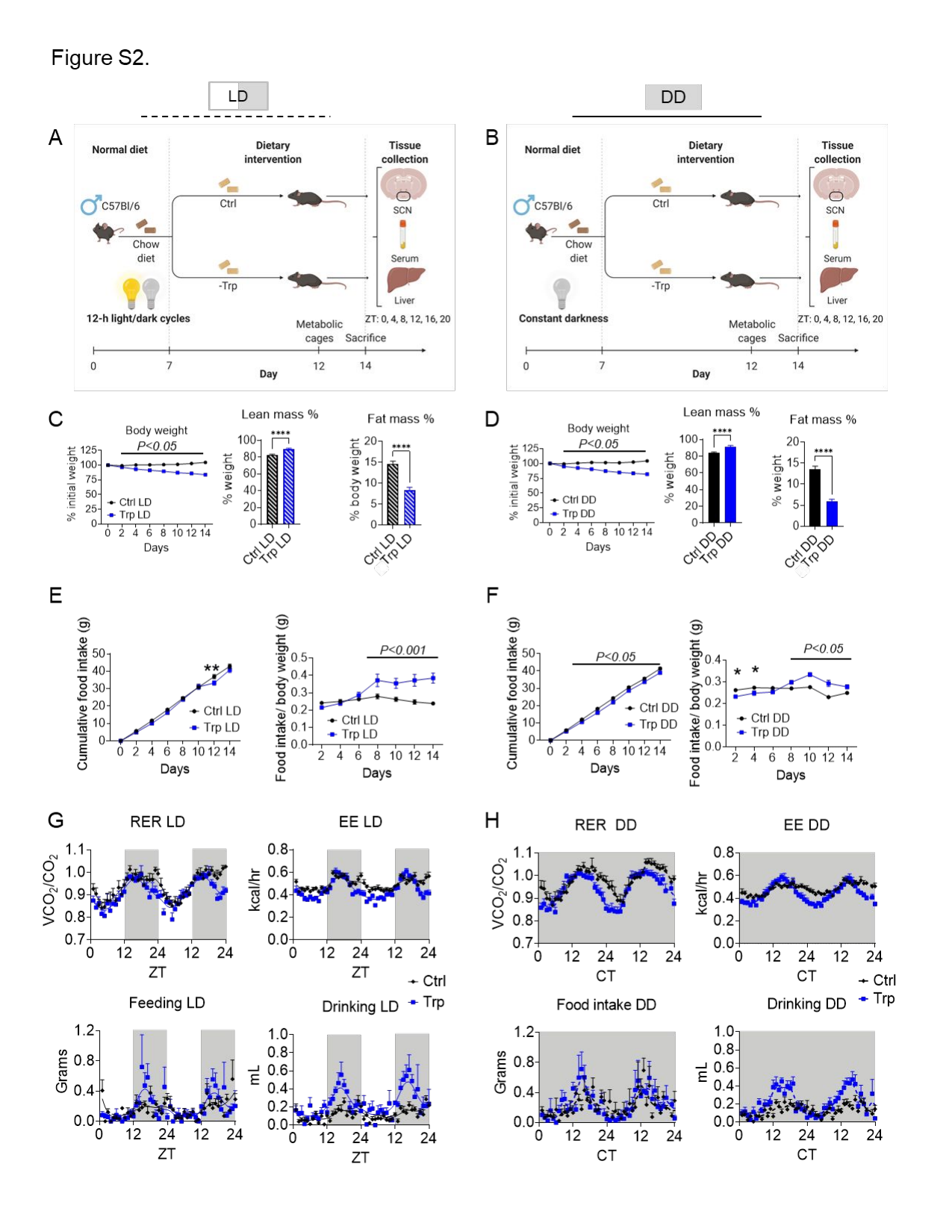

## Slide 3
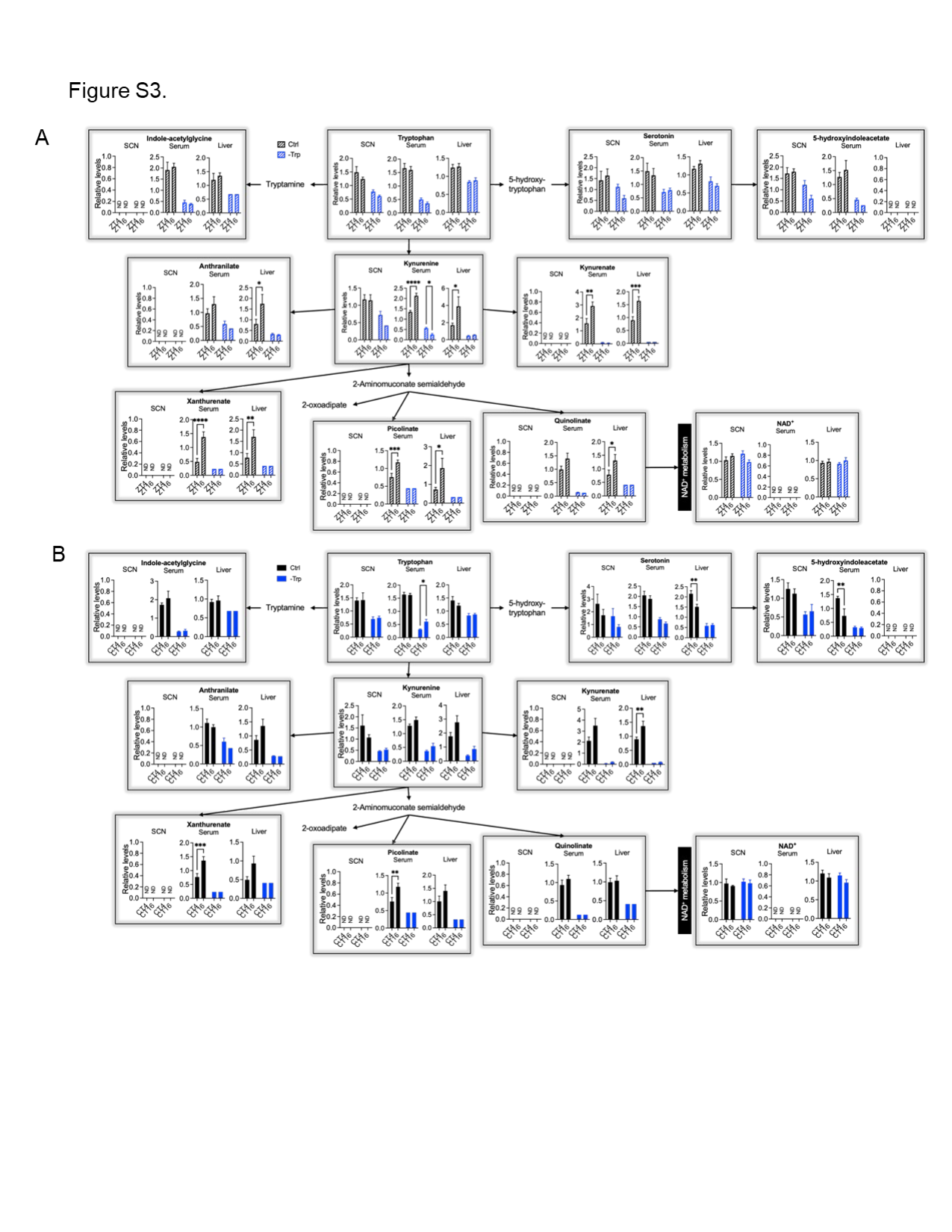

## Slide 4
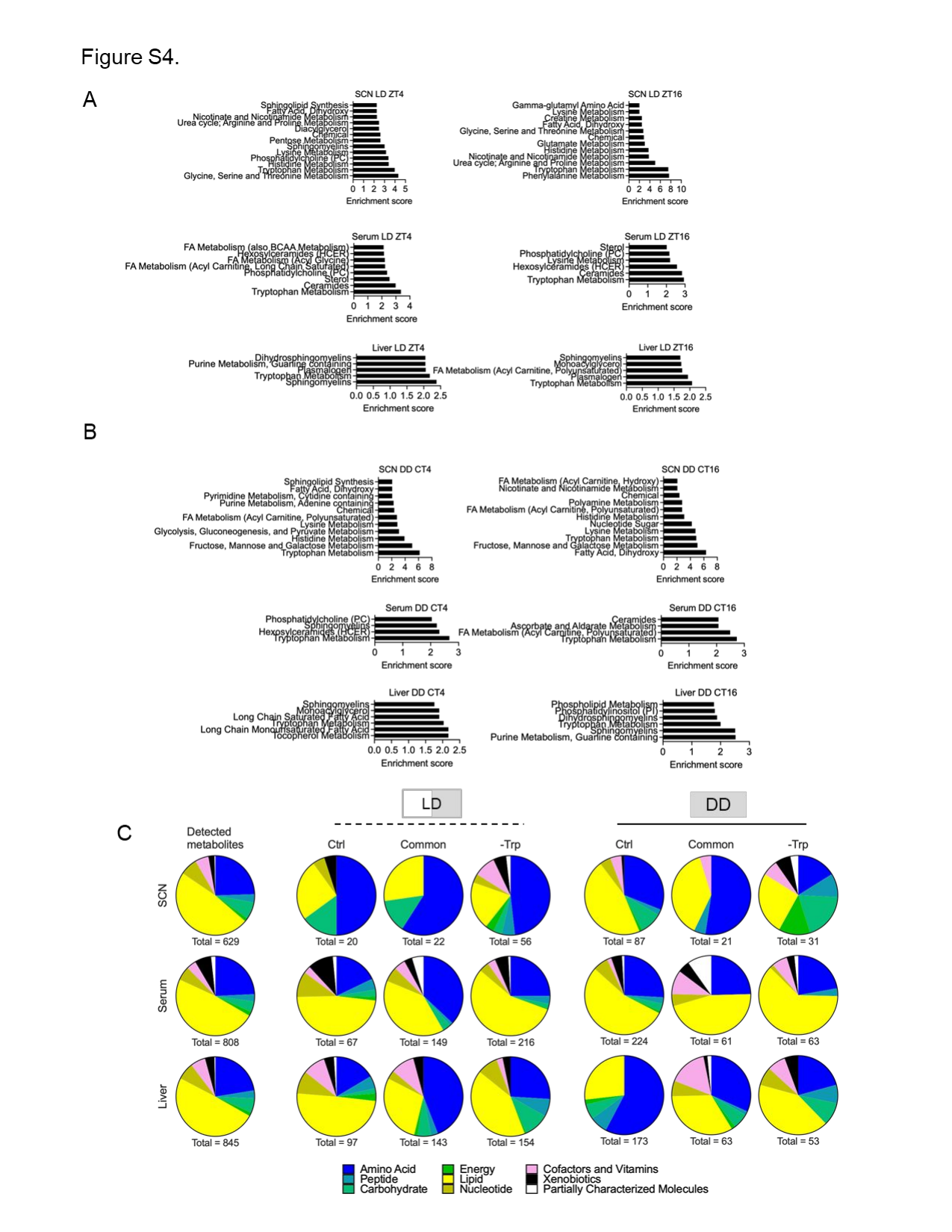

## Slide 5
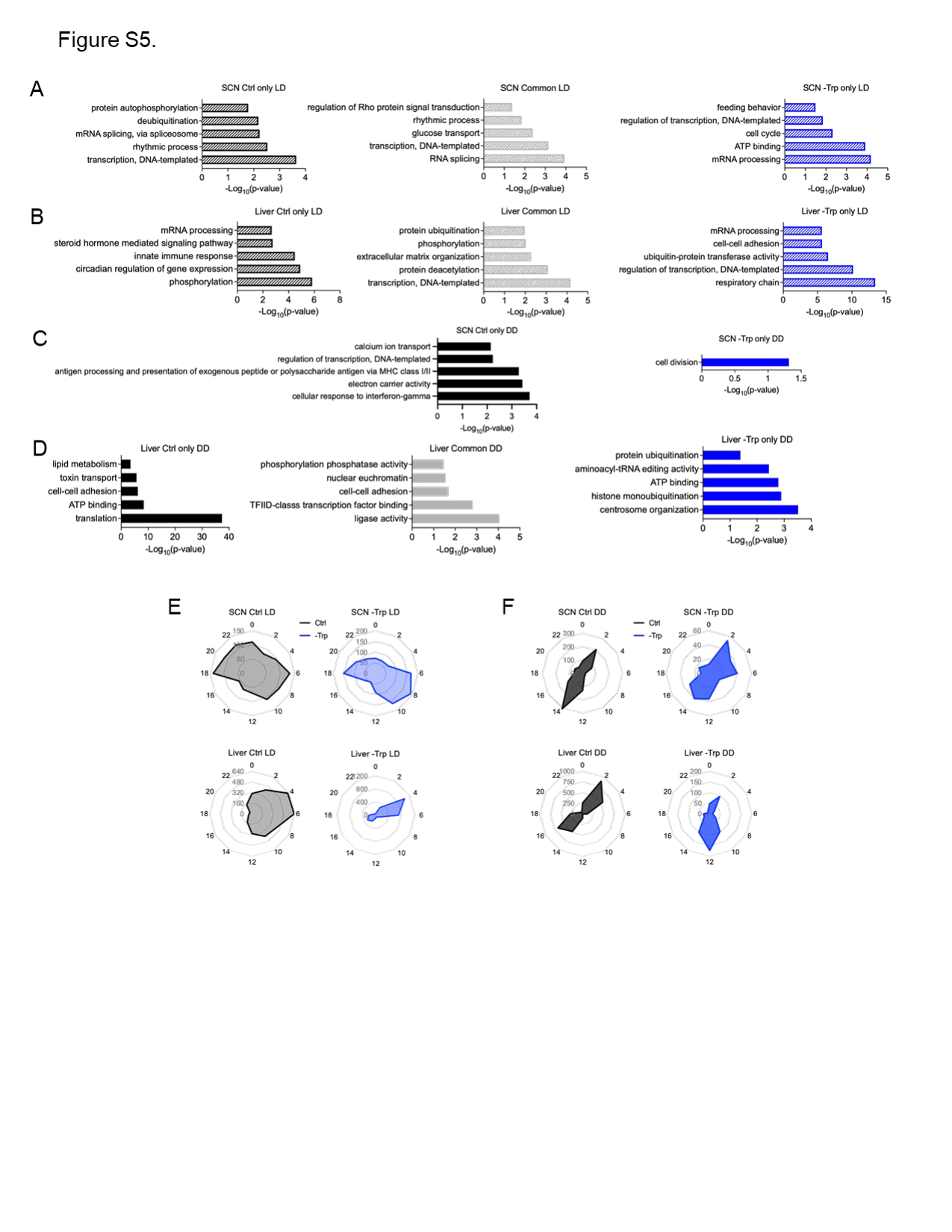

Supplement: Multimedia component 2 [file mmc2.pptx]
